# Physicochemical properties and fatty acid profile of oil extracted from black soldier fly larvae (*Hermetia illucens*)

**DOI:** 10.14202/vetworld.2024.518-526

**Published:** 2024-03-05

**Authors:** Krittika Srisuksai, Paviga Limudomporn, Uthaiwan Kovitvadhi, Khunakon Thongsuwan, Witcha Imaram, Ratchaphon Lertchaiyongphanit, Tharinee Sareepoch, Attawit Kovitvadhi, Wirasak Fungfuang

**Affiliations:** 1 Department of Zoology, Faculty of Science, Kasetsart University, Bangkok, Thailand; 2 Department of Chemistry and Center of Excellence for Innovation in Chemistry, Faculty of Science, Kasetsart University, Bangkok, Thailand; 3 Department of Chemistry, Faculty of Science, Kasetsart University, Bangkok, Thailand; 4 Department of Physiology, Faculty of Veterinary Medicine, Kasetsart University, Bangkok, Thailand

**Keywords:** black soldier fly, extraction method, fatty acid, *Hermetia illucens*

## Abstract

**Background and Aim:**

*Hermetia illucens*, a black soldier fly, is widely recognized for sustainable recycling of organic waste. Black soldier fly larvae (BSFLs) can consume various types of biowastes and convert them into nutrient-rich biomass, including proteins, lipids, chitin, and minerals. This study investigated the best extraction method by comparing the fatty acid profiles, percentage yield, and antioxidant properties of BSFL oil extracted using different extraction methods.

**Materials and Methods:**

The physicochemical properties, fatty acid profile, and free radical scavenging ability of BSFL oil were analyzed using six extraction methods.

**Results:**

Ultrasonic extraction with hexane resulted in the highest yields compared with different extraction methods. Lauric acid (28%–37%) was the most abundant fatty acid in all extracts, followed by palmitic acid, myristic acid, oleic acid, and linoleic acid. Compared with other methods, aqueous extraction showed the highest lauric acid composition and free radical scavenging activities. In addition, high-temperature aqueous extraction resulted in higher oil yield and free radical scavenging activities than low-temperature extraction.

**Conclusion:**

High-temperature aqueous extraction is the best extraction method because it is rich in lauric acid, has antioxidant ability, and can be further developed to produce novel sustainable biomaterials for humans and animals.

## Introduction

Antioxidants play an important role in free radical scavenging both *in* *vivo* and *in* *vitro* [1]. Antioxidant substances can be found in various food products, such as vegetables and oils, and their consumption can reduce the risk of chronic diseases [2]. The use of synthetic antioxidants such as butylated hydroxytoluene (BHT), butylated hydroxyanisole, propyl gallate, and citric acid in food processing has recently led to an increased occurrence of side effects (e.g., carcinogenesis) [3]. Thus, there has been an increase in the number of studies focusing on natural antioxidants over the past decade [4]. The black soldier fly, *Hermetia* *illucens* L. (*Diptera*: *Stratiomyidae*), specifically the black soldier fly larvae (BSFL), has received increased attention from scientists because BSFL can consume a diverse range of organic waste materials and is of nutritional benefit [5]. The nutritional composition of BSFL depends on the rearing substrate; in general, it contains 40% protein and 30% fat on a dry matter basis [6, 7]. However, nutritional biomass can vary depending on the type of growing substrate [8]. Natural oils contain various important fatty acids and are extensively used in cosmetics and skin treatments worldwide [9]. Oil quality primarily depends on its fatty acid composition [10]. Previous studies have found that BSFL oil is rich in saturated fatty acids (SFAs), mainly lauric acid [11, 12], which exhibits antioxidant, antibacterial, antiviral, antifungal, and anticancer properties [7, 13, 14]. These observations are in agreement with those of a recent study showing that BSFL oil has antioxidant activities without causing toxicity in *Artemia* *salina* [11]. Moreover, broiler chickens fed with BSFL oil exhibited improved immune and antioxidant functions in the blood plasma [15]. Fatty acid profiles, extraction yields, and antioxidant properties of oils can vary depending on the extraction method employed [16].

Conventional oil extraction processes include solvent extraction, mechanical pressing, and aqueous extraction [17]. Solvent extraction (e.g., hexane [H] and ethanol [E]) has a high oil yield and easy recovery [18]. However, the use of these organic solvents has led to increased concerns about their impact on general health and environmental protection. For example, H is released into the environment to form ozone and photochemicals [17]. H and E are readily soluble in neural lipids [17, 19]. Moreover, several studies have revealed that H and E inhalation affects the neural system. Because of these health, safety, and environmental concerns, there has been a search for substitutes for organic solvents. Other alternatives include much simpler and safer methods that involve fewer steps, such as the mechanical pressing method [20]. However, drawbacks such as relatively high energy demand and low efficiency make the mechanical pressing method less popular [21]. Aqueous extraction is an eco-friendly alternative process that eliminates the use of organic solvents and requires lower investment costs and energy demand [18]. Therefore, aqueous extraction is gaining popularity to obtain high-quality oils, such as palm oil, peanut oil, and larval oil [11, 21, 22].

Therefore, this study aimed to investigate the best extraction method by comparing the fatty acid profiles, percentage yield, and antioxidant properties of BSFL oil extracted using different extraction methods. In addition, we update the fatty acid profiles of BSFL oil. The findings of this study could be used for the production and development of dietary products and sustainable biomaterials.

## Materials and Methods

### Ethical approval

The research conducted adhered to the Guidelines for the Care and Use of Laboratory Animals. The ethics committee of Kasetsart University Research and Development Institute, Kasetsart University, Thailand, approved this study (ID: ACKU66-SCI-021).

### Study period and location

This study was conducted for eight months (November 2022 to June 2023). BSFL was cultivated on a domestic farm in Pathum Thani Province, Thailand. The physicochemical and free radical scavenging ability of BSFL oil were conducted at Department of Zoology, Faculty of Science, Kasetsart University. The fatty acid profile of BSFL oil analysis was conducted at Department of Chemistry Faculty of Science, Kasetsart University.

### Reagents

2,2-diphenyl-1-picrylhydrazyl (DPPH) and 2,2′-azino-bis (3-ethylbenzothiazoline-6-sulfonic acid) (ABTS), ethyl acetate, and ethyl alcohol were obtained from MilliporeSigma (Burlington, MA, USA) and Thermo Fisher Scientific (Waltham, MA, USA), respectively. Potassium persulfate was procured from PanReac AppliChem (Barcelona, Spain). H was purchased from Macron Fine Chemicals (Radnor, PA, USA). All reagents and chemicals used were of analytical grade.

### BSFL cultivation and preparation

BSFL was cultivated on a domestic farm in Pathum Thani Province, Thailand. Larvae were raised in 60 × 40 × 10 cm plastic containers in controlled environments (28 ± 2°C on a 12-h light/12-h dark cycle) Larvae were fed a mixture of coconut endosperm and soybean curd residue in a 50:50 ratio at a humidity of 70% [5]. The BSFL prepupa obtained from the culture was sieved and washed to remove any feed. The BSFL preparations were then frozen and kept at –20°C until the experiment was performed.

Samples were prepared using the method described by Krzyżaniak *et al*. [23]. Briefly, to stop the browning reaction, the frozen BSFL prepupa was blanched in boiling water for 35 s. Subsequently, the larvae were thinly spread on an aluminum tray (500 g larvae per tray) and baked under convective hot air. Baking was performed at 65°C for 6 h to ensure that the larvae achieved full dryness and were subsequently crushed in a grinder. Crushed larvae were then placed in Ziploc bags and stored at 20°C until use.

### Oil extractions

#### Aqueous extraction using a magnetic stirrer

Aqueous extraction with a magnetic stirrer was performed based on the method described by Yashashri *et al*. [24] with slight modifications. In brief, 70 g of crushed larvae was extracted with 195 mL of distilled water in a beaker with a magnetic stirrer for 15 min. This setup was run at 45°C (M45), 65°C (M65), and 95°C (M95). The extract was centrifuged at 2200× *g* for 15 min. The lipid layer formed at the top was collected, weighed, and stored at room temperature until further analysis.

#### Mechanical pressing and extraction

Mechanical pressing extraction (M) was performed based on the method described by Rabadán *et al*. [25] with slight modification; extraction was performed using a Stainless Steel SUS304 single screw press (Siam Wealth Food, Co. Ltd., Nonthaburi, Thailand). The dried BSFL was pressed using a rotating screw press at an extraction temperature of 150°C. The maximum machine capacity load was 5 kg/h. The extracts were collected, weighed, and stored at room temperature for further analysis.

#### Ultrasonic extraction using an organic solvent

Ultrasonic extraction with organic solvents was performed according to the method described by Almeida *et al*. [11] with slight modifications. Extraction was performed using n-H and E organic solvents. Crushed larvae (70 g) in 195 mL of solvent were subjected to a 15-min cold ultrasonic treatment (Cole-Parmer, Vernon Hills, IL, USA) before timed agitation. Subsequently, the extract was centrifuged at 2200× *g* for 15 min. We collected the liquid layer at the top and removed the solvent on a rotary vacuum evaporator (R-215, Buchi, Bangkok, Thailand) at 50°C. The obtained extracts were collected, weighed, and stored at room temperature for further analysis.

### Physicochemical properties

#### Extraction yield (%)

The amount of oil extracted through each method was weighed to calculate the extraction yield using Equation 1.

Extraction yield (%) = (Oil weight/Sample weight) × 100 (1)

### Color intensity

The color intensities of the oil samples were determined using an Ultraviolet–vis spectrophotometer (Spark™ 10M, TECAN, Mannedorf, Switzerland). Oil was placed in a 96-well plate, and absorbance was recorded at a wavelength of 425 nm. Each sample was analyzed in triplicate.

### Fatty acid composition

Following the method of Buthelezi *et al*. [26] with slight modifications; Fatty acid methyl ester (FAME) analysis was performed using a gas chromatography-mass spectrometer (GCMS-QP2020, Shimadzu, Kyoto, Japan) with a fatty acid methyl ester column (ZB-FAME, Zebron, Newport Beach, CA, USA) with a length of 30 m, an internal diameter of 0.25 mm, and a stationary phase film thickness of 0.20 μm to determine the fatty acid composition in the BSFL oil. One μL of the derivatized sample was injected in split mode at a split ratio of 1:50 using He (99.999%) as the carrier gas at a constant flow rate of 1 mL/min. The injector and interface temperatures were set to 240°C and 260°C, respectively. The oven temperature was programmed from 40°C to 150°C, then 2°C/min to 170°C, and held for 7 min, then ramped at a rate of 10°C/min to 200°C, increased to 240°C at 20°C/min, and kept again for 2 min to elute any remaining impurities. The total run time was 35 min, and the fatty acids were identified by comparing their retention times with those of authentic standard fatty acid methyl esters. An electron ionization system with an ionizing energy of 70 eV and an ion source at 240°C was used for GC/MS detection.

### Free radical scavenging activity of the BSFL oil

#### DPPH radical scavenging assay

The DPPH assay was performed on the basis of the method described by Srisuksai *et al*. [27] with slight modifications. If a hydrogen atom or electron is transferred to the DPPH radical (DPPH), then the absorbance at 517 nm decreases proportionally to the increase in the number of non-radical forms of DPPH. Briefly, 50 μL of each BSFL oil extracted using differential methods was added to 150 μL of DPPH in ethyl acetate (150 μM). The absorbance at 517 nm decreased proportionally with an increase in the number of non-radical particles. After vortex mixing, the mixture was incubated at room temperature for 30 min, and absorbance was measured at 517 nm. BHT was used as a positive control, and all samples were tested in triplicate. We documented the differences in the absorbance of each oil, BHT, and control (DPPH only). Radical scavenging activity was calculated as the percentage inhibition using the following equation:

% inhibition = [(control test)/control] × 100 (2)

The half-maximal inhibitory concentration (IC_50_) was determined as the oil concentration that elicited a 50% decrease in absorbance compared with the control.

#### ABTS radical scavenging assay

The ABTS assay was performed on the basis of the method described by González-Palma *et al*. [28], with slight modifications. This assay is based on decolorization, which occurs when the radical cation ABTS+ is reduced to ABTS. Briefly, 7 mM ABTS solution and 2.45 mM potassium persulfate solution were used as the stock solutions. The working solution was then prepared by mixing equal quantities of the two stock solutions and allowed to react for 16 h at room temperature in the dark. After incubation, the solution was further diluted at water: E ratio of (1:1) until an initial absorbance value of 0.7 0.1 at 734 nm was achieved. Fresh ABTS solution was prepared for each assay. BSFL oil extracted using various methods (20 μL) was allowed to react with 180 μL of the ABTS solution, and absorbance was measured at 734 nm after 60 min using a spectrophotometer. BHT was used as a positive control, and all samples were tested in triplicate. We documented the differences in the absorbance of BSFL oil extracted using various methods, BHT, and control (ABTS only). Radical scavenging activity was calculated as the percentage inhibition using the following equation:

% inhibition = [(control test)/control] × 100 (3)

The half-maximal IC_50_ was defined as the concentration of oil that elicited a 50% decrease in absorbance compared with the control.

### Statistical analysis

Statistical analyses were performed using the R Statistical Software (v4.1.2; R core team, 2021; https://www.r-project.org/) [29]. Data are presented as mean standard deviation. All data were analyzed using a one-way analysis of variance followed by Tukey’s *post hoc* test. A p = 0.05 was considered statistically significant.

## Results

### Physicochemical properties of the BSFL oil

**Figure-1:**
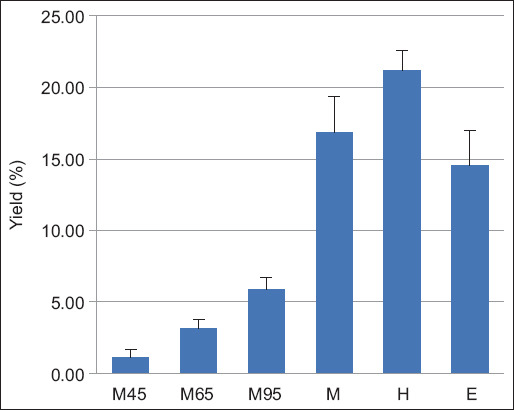
Yield obtained from different extraction methods. Values with a different letter above the bar differ significantly (p < 0.05). M45=aqueous extraction with a magnetic stirrer at the temperature of 45°C, M65=Aqueous extraction with a magnetic stirrer at the temperature of 65°C, M95=Aqueous extraction with a magnetic stirrer at temperature of 95°C, M=Mechanical pressing extraction, H=Ultrasonic extraction with hexane, E=Ultrasonic extraction with ethanol.

The percentage yields of BSFL oil obtained through various aqueous extractions with magnetic stirrers (M45, M65, and M95), M, ultrasonic extraction with H, and E were 1.11 ± 0.55, 3.15 ± 0.63, 5.86 ± 0.90, 16.82 ± 2.58, 21.19 ± 1.40, and 14.52 ± 2.46, respectively (Figure-1). When compared with the other extraction methods used in this study, extraction with H gave the highest yield.

**Figure-2:**
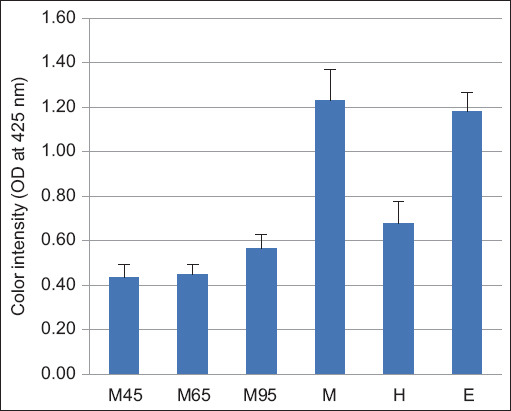
Color intensity of BSFL oils extracted using various methods. Values with a different letter above the bar differ significantly (p < 0.05). M45=Aqueous extraction with a magnetic stirrer at temperature of 45°C, M65=Aqueous extraction with a magnetic stirrer at temperature of 65°C, M95=Aqueous extraction with a magnetic stirrer at temperature of 95°C, M=Mechanical pressing extraction, H=Ultrasonic extraction with hexane, E=Ultrasonic extraction with ethanol, BSFL=Black soldier fly larvae.

The color intensity after BSFL oil extraction using various methods indicated that the extracted samples exhibited a yellow color when compared with the blank value (Optical density = 0.0480). The yellow color intensity of the oil obtained using the M and E methods was significantly higher than that obtained using the M45, M65, M95, and H methods (p < 0.05; Figure-2).

### Fatty acid profiles of BSFL oil

Fatty acid profiles of BSFL oil extracted using the E method had significantly higher levels of total SFAs than those extracted using the M45, M65, M95, M, and H methods (p < 0.05; Table-1). In addition, oil extracted using the E method had significantly lower levels of total monounsaturated fatty acids (p < 0.05; Table-1). However, the BSFL oil extracted using the E method did not have a total polyunsaturated fatty acid (PUFA) content. The total PUFA content in BSFL oil extracted using the M95 (13.81 ± 0.60%) and M (12.97 ± 0.85%) methods was significantly higher (p < 0.05) than that extracted using the M65 (9.36 ± 1.58%). The unsaturated fatty acid (UFA)/SFA ratio in BSFL oil extracted by the M95 (0.48 ± 0.01), M (0.48 ± 0.01), and H (0.45 ± 0.03) methods was significantly higher (p < 0.05) than that extracted by the M65 (0.36 ± 0.05%) and E (0.01 ± 0.00) methods (p = 0.05).

**Table-1:** Fatty acid composition of BSFL oil analyses through gas chromatography.

Fatty acids	Fatty acid composition (%)					

	M45	M65	M95	M	H	E
Capric acid (C10:0)	1.01 ± 0.04^b^	1.00 ± 0.06^b^	0.92 ± 0.05^bc^	0.80 ± 0.06^c^	0.86 ± 0.03^bc^	1.25 ± 0.07^a^
Lauric acid (C12:0)	34.13 ± 0.47^ab^	32.93 ± 2.78^bc^	30.88 ± 1.32^bcd^	28.39 ± 2.17^d^	29.44 ± 0.74^cd^	37.82 ± 1.03^a^
Tridecanoic acid (C13:0)	0.12 ± 0.07	0.05 ± 0.01	0.04 ± 0.00	0.04 ± 0.01	0.07 ± 0.05	0.06 ± 0.00
Myristic acid (C14:0)	15.81 ± 0.75^b^	16.13 ± 1.16^b^	14.22 ± 0.45^bc^	12.46 ± 0.61^c^	13.57 ± 0.32^c^	20.50 ± 0.83^a^
Pentadecanoic acid (C15:0)	0.15 ± 0.01^cd^	0.16 ± 0.01^bc^	0.14 ± 0.00^d^	0.18 ± 0.01^b^	0.15 ± 0.01^cd^	0.23 ± 0.01^a^
Palmitic acid (C16:0)	15.80 ± 1.01^b^	16.54 ± 0.96^b^	14.65 ± 0.55^b^	16.73 ± 0.74^b^	15.91 ± 0.31^b^	23.09 ± 0.98^a^
Margaric acid (C17:0)	-	0.14 ± 0.00^ab^	0.13 ± 0.01^b^	0.20 ± 0.03^ab^	0.18 ± 0.07^ab^	0.26 ± 0.08^a^
Stearic acid (C18:0)	2.88 ± 0.22^d^	3.07 ± 0.15^cd^	2.75 ± 0.09^d^	3.95 ± 0.15^b^	3.33 ± 0.04^c^	4.40 ± 0.18^a^
Nonadecanoic acid (C19:0)	-	-	-	-	0.09 ± 0.01	-
Total SFA	69.90 ± 1.55^b^	70.01 ± 5.10^b^	63.72 ± 2.44^b^	62.75 ± 3.71^b^	63.52 ± 1.30^b^	87.62 ± 3.14^a^
Palmitoleic acid (C16:1)	1.44 ± 0.15^a^	1.10 ± 0.15^ab^	1.44 ± 0.24^a^	0.75 ± 0.03^b^	0.89 ± 0.27^b^	-
Petroselinic acid (C18:1 *n*-12)	1.26 ± 0.28^cd^	1.83 ± 0.46^ab^	1.14 ± 0.20^cd^	2.54 ± 0.21^a^	1.74 ± 0.25^ab^	0.57 ± 0.22^d^
Oleic acid (C18:1 *n*-9)	14.48 ± 0.96	12.78 ± 1.04	14.10 ± 1.32	13.66 ± 0.36	13.66 ± 0.97	-
Total MUFA	16.76 ± 0.40^a^	15.71 ± 0.83^a^	16.69 ± 1.38^a^	16.95 ± 0.60^a^	16.29 ± 0.48^a^	0.57 ± 0.22^b^
Linoleic acid (C18:2 *n*-6)	10.17 ± 0.93^ab^	8.61 ± 1.40^b^	11.89 ± 1.54^a^	11.84 ± 0.80^ab^	11.08 ± 1.22^ab^	-
alpha-Linolenic acid (C18:3 *n*-3)	0.87 ± 0.16^ab^	0.75 ± 0.18^b^	1.24 ± 0.23^a^	1.13 ± 0.06^ab^	1.06 ± 0.16^ab^	-
Total PUFA	11.04 ± 1.09^ab^	9.36 ± 1.58^b^	13.81 ± 0.60^a^	12.97 ± 0.85^a^	12.14 ± 1.37^ab^	-
Other	2.30 ± 0.16	4.91 ± 3.97	6.46 ± 5.51	7.34 ± 5.08	8.05 ± 2.76	11.81 ± 3.30
UFA/SFA	0.40 ± 0.03^ab^	0.36 ± 0.05^b^	0.48 ± 0.01^a^	0.48 ± 0.01^a^	0.45 ± 0.03^a^	0.01 ± 0.00^c^

Values in the same row with different superscript letters differ significantly (p < 0.05). M45=Aqueous extraction with a magnetic stirrer at temperature of 45°C, M65=Aqueous extraction with a magnetic stirrer at temperature of 65°C, M95=Aqueous extraction with a magnetic stirrer at temperature of and 95°C, M=mechanical pressing extraction, H=Ultrasonic extraction with hexane, E=Ultrasonic extraction with ethanol, PUFA=Polyunsaturated fatty acid, MUFAs=Monounsaturated fatty acid, SFA=Saturated fatty acid, UFA=Unsaturated fatty acid

### Free radical scavenging activity of BSFL oil using the DPPH assay

**Figure-3:**
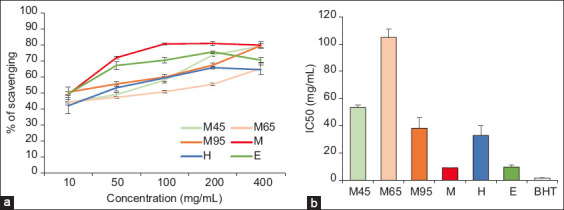
Free radical scavenging of BSFL oils extracted using various methods using the DPPH assay. (a) Free radical scavenging ability. (b) IC_50_ of BSFL oil extracted by M45, M65, M95, M, H, E, and BHT. Values are presented as mean SD. M45=Aqueous extraction with a magnetic stirrer at temperature of 45°C, M65=Aqueous extraction with a magnetic stirrer at temperature of 65°C, M95=Aqueous extraction with a magnetic stirrer at temperature of 95°C, M=Mechanical pressing extraction, H=Ultrasonic extraction with hexane, E=Ultrasonic extraction with ethanol, BSFL=Black soldier fly larvae, DPPH=2,2-diphenyl-1-picrylhydrazyl.

Figure-3 presents the free radical scavenging activity of the BSFL oil extracted using different methods. Oil extracted using the M45, M65, M95, M, H, and E methods showed a dose-dependent inhibitory effect on DPPH free radical scavenging ability (Figure-3a). The IC_50_ (in mg/mL) of BSFL oil extracted using the M45, M65, M95, M, H, and E methods, including BHT, was 53.28 ± 1.88, 104.90 ± 6.11, 38.24 ± 7.79, 9.06 ± 0.16, 32.89 ± 7.14, 9.59 ± 1.64, and 1.53 ± 0.08, respectively (Figure-3b). The DPPH free radical scavenging ability of the BSFL oils extracted using different methods and BHT was in the order BHT > M > E > H > M95 > M45 > M65.

### Free radical scavenging activity of BSFL oil using the ABTS assay

**Figure-4:**
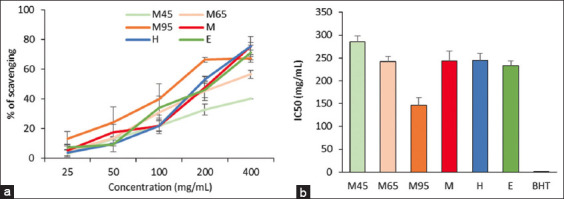
Free radical scavenging of BSFL oils extracted using various methods using the ABTS assay. (a) Free radical scavenging ability. (b) IC_50_ of BSFL oil extracted by M45, M65, M95, M, H, E, and BHT. Values are presented as mean SD. M45=Aqueous extraction with a magnetic stirrer at temperature of 45°C, M65=Aqueous extraction with a magnetic stirrer at temperature of 65°C, M95=Aqueous extraction with a magnetic stirrer at temperature of 95°C, M=Mechanical pressing extraction, H=Ultrasonic extraction with hexane, E=Ultrasonic extraction with ethanol, BSFL=Black soldier fly larvae, ABTS=2,2′-azino-bis(3-ethylbenzothiazoline-6-sulfonic acid), SD=Standard deviation.

Figure-4 presents the free radical scavenging activity of the BSFL oil extracted using different methods. A dose-dependent inhibitory effect on the ability to scavenge ABTS free radicals was observed for BSFL oil extracted using the M45, M65, M95, M, H, and E methods (Figure-4a). The IC_50_ (in mg/mL) of BSFL oil extracted using the M45, M65, M95, M, H, and E methods, including BHT, was 285.41 ± 13.46, 242.17 ± 11.88, 146.58 ± 17.06, 243.04 ± 21.54, 244.87 ± 14.77, 233.51 ± 10.07, and 0.19 ± 0.02, respectively (Figure-4b). The ability of BSFL oils to scavenge ABTS free radicals was in the order BHT > M95 > E > M65 > M > H > M45.

## Discussion

The black soldier fly is one of the most interesting insects because of its ability to bioconvert organic wastes, such as agricultural waste, animal farm waste, and house organic waste, into high biomass and sustainable biological products. Insect production is inexpensive and environmentally friendly, given several feeding sources, high land-use efficiency, and reduction of greenhouse gasses [30]. Previous studies have indicated that BSFL can reduce biomass by 30%–50%, thereby converting it into high-quality nutrients that can be used as feed for domestic animals, exotic foods, and fish and poultry [31, 32].

In the present study, we focused on the BSFL oil extraction method and demonstrated that it affected both the percent extraction yield and the physicochemical properties of the oil. Organic solvents commonly used for lipid extraction include H, 3:2 H-isopropanol, chloroform, 2:1 chloroform-methanol, and methyl-tert-butyl-ether [33], depending on the sample polarity. Organic solvents can extract different classes of compounds according to their polarity [11]. This explains the higher extraction percentage for H and E compared with the other extraction methods used in this study (Figure-1). In addition, oil extraction with H yielded higher yields than that with E (Figure-1). H has a lower polarity than E [34]. As the polarity of the solvent decreases, the oil extraction yield increases [35]. This finding is in agreement with previous studies in which oil extracted by hexane gave the highest yield [11, 36, 37].

The BSFL oil obtained using the M and E methods had a yellow color higher than that obtained using the M45, M65, M95, and H methods (Figure-2). Hydraulic compression-producing oil (M method) showed a yellow cloudy oil, which may be due to the pigment content [38, 39]. The high polarity of E is capable of extracting nonglyceride components such as phosphatides, sterols, tocopherols, and pigments [40]. This finding is in agreement with previous studies in which E-based solvents appear slightly more colored [41, 42]. Similar to a previous study by Wu *et al*. [43], our study found that the color of the BSFL oil extracted by the aqueous method is clear yellow. In addition, the clear yellow of oil extracted by the H method is most likely due to bleaching [39].

Our results showed that SFAs are the major component of all BSFL oil extracts (Table-1). Among the SFAs, lauric acid had the highest abundance, in agreement with previous reports [32, 44], with the highest yield obtained using E extraction. UFAs were almost absent in the BSFL oil extracted with E (Table-1). A rotary evaporator was used to separate the oil from the solvents in ultrasonic extraction with organic solvents. The vacuum pressure for the rotary evaporation of E is lower than that for H. The intermolecular attraction of UFAs is weaker than that of SFAs, leading to a lower boiling point [45]. Therefore, UFAs may evaporate to the bottom of the solvent, leaving only SFAs in the BSFL oil extracted with E. Fatty acid profiles were present in all BSFL oil extracts in the following order: Lauric acid, palmitic acid, myristic acid, oleic acid, and linoleic acid [11, 32, 44]. Similar to a previous study by Almeida [11] and He *et al*. [46], our study demonstrated that the SFAs in aqueous extraction were higher than those in mechanical and H extraction. Different fatty acid compositions may be due to oxidation during the extraction process, extraction techniques, and the fatty acid structure [39]. In addition, previous studies have suggested that the extraction method temperature affects the fatty acid concentration [47, 48]. High extraction temperatures could trigger the degradation of free fatty acids into volatile components [48]. Similar to a previous study by Mohamed *et al*. [49], our study found that increasing temperature reduced the concentration of lauric acid. However, the fatty acid profile of the BSFL oil also depends on the substrate used to rear the BSFL [50, 51]. BSFL reared with fermented maize stover has the lowest lauric acid content, whereas BSFL reared with vegetable and fruit waste has the highest lauric concentration [32]. Other studies have indicated that a high concentration of carbohydrates in foods can increase the lauric acid content of BSFL oil, suggesting that BSFL can metabolize carbohydrates into lauric acid [6, 51].

The ability of BSFL oil to scavenge free radicals was determined using DPPH and ABTS *in vitro* antioxidant assays. Similar to a previous study by Phongpradist *et al*. [52], our results indicated that BSFL oil could scavenge free radicals [52]. In both the DPPH and ABTS assays, the BSFL oil extracted with E had a higher free radical scavenging ability than the oil extracted using other methods (Figures-3 and 4). E is highly capable of extracting nonglyceride components such as phosphatides, sterols, tocopherols, and pigments [40]. Previous studies by Dos Santos Aquilar [53] have shown that tocopherols in insect larval oil may contribute to antioxidant capacity by acting as a chain-breaking agent. In addition, compared with the other methods, BSFL oil extracted by mechanical pressing showed the highest free radical scavenging ability in the DPPH assay (Figure-3). To obtain natural oil products, the pressing method is based only on a mechanical process [54]. Therefore, the pressing method is a rich source of phytosterols, tocopherols, carotenoids, and polyphenols that are associated with antioxidant capacity [55, 56]. According to previous studies, the pressing method has advantages with regard to the retention of active components to scavenge free radicals both *in vivo* and *in vitro* [57, 58]. However, aqueous extraction of BSFL oil at 95°C resulted in higher free radical scavenging in both DPPH and ABTS assays compared with aqueous extraction at 45°C and 65°C (Figures-3 and 4). An earlier study demonstrated that an increase in water temperature reduces surface tension and viscosity, which increases the diffusion and mass transfer rates during extraction [59]. Previous studies have demonstrated that aqueous extraction with boiling water contains more antioxidant compounds than cool water [60, 61]. Similar to our study, a previous study by Thuanthong *et al*. [59] found that increasing the extraction temperature from 70°C to 80°C leads to higher DPPH and ABTS radical scavenging activities [59]. The DPPH scavenging activity was found to be higher than the ABTS cation scavenging activity (Figures-3 and 4). Stereoselectivity of the radicals or solubility of the extract in different testing systems could affect the capacity of extracts to react and scavenge different radicals [62, 63].

Most researchers are looking for new sources of proteins, lipids, or sustainable biological resources. A high concentration of lauric acid in the BSFL oil indicates its potential use, which is similar to that of coconut oil and palm kernel oil [36]. Several studies have shown that lauric acid has antibacterial, antiviral, antifungal, and anticancer activities [7, 13, 14]. Moreover, lauric acid is widely used as a raw material for the production of surfactants in the food, cosmetic, shampoo, and pharmaceutical industries [7]. Palmitic, myristic, oleic, and linoleic acids constituted the fatty acid profile of the BSFL oil. Several studies have indicated the beneficial properties of fatty acids, such as myristic acid, which can function as a skin penetration enhancer [64] and are used as a cosmetic ingredient [32], oleic acid, which regulates epidermal lipid metabolism [64], palmitic acid and its derivatives as emollients, and linoleic acid, which contributes to the water impermeability of the stratum cornel [11].

## Conclusion

Although ultrasonic extraction using organic solvents (H and E) yielded the highest oil yields, health and the environment are of concern. However, aqueous extraction yielded the highest amount of lauric acid compared with other methods. Interestingly, aqueous extraction at a high temperature (M95) yielded higher oil yields and free radical scavenging activities compared with lower temperatures (M45 and M65). Therefore, we conclude that high-temperature aqueous extraction is the best extraction method in this study. The quality of the BSFL oil thus obtained can ensure its use as a sustainable bionutrient, biomedical material, and pharmaceutical substance. Further studies are required to increase the effectiveness of the extraction methods discussed and the purity, quality, and quantity of BSFL oil.

## Authors’ Contributions

KS: Performed the experiment, analyzed the data, and wrote the manuscript. PL, UK, KT, and AK: Performed the experiment and edited the manuscript. WI, RL, and TS: Provided and analyzed fatty acid profile and edited the manuscript. WF: Conceived, designed and supervised the study and edited the manuscript. All authors have read, reviewed, and approved the final manuscript.
